# Variable Importance and Prediction Methods for Longitudinal Problems with Missing Variables

**DOI:** 10.1371/journal.pone.0120031

**Published:** 2015-03-27

**Authors:** Iván Díaz, Alan Hubbard, Anna Decker, Mitchell Cohen

**Affiliations:** 1 Department of Biostatistics, Johns Hopkins Bloomberg School of Public Health, Baltimore, MD, USA; 2 Division of Biostatistics, University of California, Berkeley, CA, USA; 3 Department of Surgery, University of California San Francisco, San Francisco, CA, USA; Banner Alzheimer’s Institute, UNITED STATES

## Abstract

We present prediction and variable importance (VIM) methods for longitudinal data sets containing continuous and binary exposures subject to missingness. We demonstrate the use of these methods for prognosis of medical outcomes of severe trauma patients, a field in which current medical practice involves rules of thumb and scoring methods that only use a few variables and ignore the dynamic and high-dimensional nature of trauma recovery. Well-principled prediction and VIM methods can provide a tool to make care decisions informed by the high-dimensional patient’s physiological and clinical history. Our VIM parameters are analogous to slope coefficients in adjusted regressions, but are not dependent on a specific statistical model, nor require a certain functional form of the prediction regression to be estimated. In addition, they can be causally interpreted under causal and statistical assumptions as the expected outcome under time-specific clinical interventions, related to changes in the mean of the outcome if each individual experiences a specified change in the variable (keeping other variables in the model fixed). Better yet, the targeted MLE used is doubly robust and locally efficient. Because the proposed VIM does not constrain the prediction model fit, we use a very flexible ensemble learner (the SuperLearner), which returns a linear combination of a list of user-given algorithms. Not only is such a prediction algorithm intuitive appealing, it has theoretical justification as being asymptotically equivalent to the oracle selector. The results of the analysis show effects whose size and significance would have been not been found using a parametric approach (such as stepwise regression or LASSO). In addition, the procedure is even more compelling as the predictor on which it is based showed significant improvements in cross-validated fit, for instance area under the curve (AUC) for a receiver-operator curve (ROC). Thus, given that 1) our VIM applies to any model fitting procedure, 2) under assumptions has meaningful clinical (causal) interpretations and 3) has asymptotic (influence-curve) based robust inference, it provides a compelling alternative to existing methods for estimating variable importance in high-dimensional clinical (or other) data.

## Introduction

Modern medical care is awash in a sea of data. The advent of new monitors, better diagnostics, electronic medical record keeping and the ideal of the quantified self has resulted in patients who are more completely measured than at any other time in medical history. While purported to allow for more complete evaluation and diagnosis our current data intense medical environment often leaves clinicians overwhelmed by the dimensionality and quantity of data. Indeed, despite the current ability to continuously measure and track multiple physiologic, demographic and biologic variables measure a patient’s clinical history in detail, clinicians still make care decisions based on a few non relational variables (they can keep in their) head combined with rules of thumb and clinical gestalt. Medical decisions therefore fail to take into consideration the possible intricate relations between all the patient’s underlying factors squandering data and missing important prognostic relationships. Fortunately, advances in the fields of biostatistics and bioinformatics have developed mathematical and computational tools that can allow optimal care decisions based on the entirety of the patient’s data, which are beyond the computational ability of the clinician at the bedside.

A primary goal in evidence-based medicine is to design and implement prognosis tools that take into account an extremely large set of measured characteristics in order to predict a patient’s most likely medical outcome. An equally important goal is to establish at each given time point which of these numerous measured characteristics is decisive in the development of the predicted outcome. These two goals have been traditionally called prediction and variable importance analysis, respectively. In addition to understanding the underlying biological mechanisms related to positive medical outcomes, the joint use of these tools can help doctors devise the optimal treatment plan according to the specific characteristics of the subject, simultaneously taking into account hundreds of variables collected for each patient.

Because of the large number of variables and the complexity of the relations between them, prediction and variable importance would be impossible to achieve without the use of complex statistical algorithms accompanied by powerful computers able to carry out a large number of computations. Such methods are aimed at helping doctors make the better treatment decisions using real time data.

Prediction and variable importance are different goals whose optimal achievement requires the use of different tools. The objective of a prediction algorithm is to accurately predict the outcome, whereas the objective for estimating variable importance measures (VIM) is to estimate the degree to which changes in the outcome are caused by changes in each of the predictor variables; VIM’s optimally supply doctors with tools for making treatment decisions [1]. This difference between prediction and VIM has two main consequences. First, VIM problems are of a *causal* nature, whereas prediction problems do not try to distinguish the relative contribution of variables to the variation observed in an outcome. Second, in order to help the decision making process, VIM parameters should have, as much as possible, a clinically relevant interpretation, such as the expected change in the outcome under a given intervention. As explained below, a meaningful interpretation can only be obtained through an intelligible characterization of VIM as a statistical (or causal) parameter defined as a mapping from a tenable (thus usually very large) statistical model into simple low-dimensional parameter(s) (a Euclidean space, e.g., differences in “adjusted” means).

Current practice in biostatistics and bioinformatics involves the use of machine learning algorithms for prediction. However, measures of VIM have been via estimated coefficients, for instance, in a penalized regression (e.g., LASSO [2]). Alternatively, measures of VIM (such as returned by random forest or neural nets [3–6] are not necessarily specific to a particular model fitting routine, but are related to the added fit (or reduced estimated risk) of a “full” model versus one without the variable. Those based on procedures like LASSO are based on arbitrary statistical models (e.g., linear with main effects), so the model is likely to misspecified and thus the interpretation of the resulting coefficients problematic. On the other hand, consider the case of regression and classification trees (e.g., random forests), where the VIM for a variable *X* is defined as the difference between prediction error when *X* is perturbed versus the prediction error otherwise [3,7]. Though such a measure does not require a relatively simple parametric model, the clinical relevance of this quantity as a measure of VIM is unclear because: 1) it does not represent a statistical or causal parameter, 2) it does not have a potential interpretation in terms of the mechanistic process that generates the data, and thus 3) the translation of the estimate into the impact of potential intervention is problematic. As an example of the technical difficulties arising from this practice, [6] discuss the “bias” of random forest VIM measures, missing the fact that bias can only be defined in terms of a statistical parameter, which is never specified in the random forest VIM analysis. We present a new VIM that 1) puts no constraints on the statistical model, so that the resulting estimate is relatively unbiased, 2) has an interpretation that is analogous to a “slope” (change in a mean for a change in the variable of interest, and 3) under further assumptions has a “causal” interpretation of great clinical relevance. Thus, it provides the virtue of more accessible regression coefficients, but is appropriate for the new powerful machine learning technology for creating more accurate diagnostic predictors.

Previous work has taken the approach we outline here [1, 8]. Whereas that work accomplished the goals of a causal VIM in a semiparametric model, the predictors were required to be discrete, preferably binary. Defining VIM parameters in terms of potential causal associations for “continuous” variables poses additional technical challenges. When researchers using causal inference methods are faced with exposures of continuous nature, the most common approach is to dichotomize the continuous exposure and consider the effect of its binary version on the outcome. This approach suffers from various flaws. First, the causal parameter does not answer questions about plausible modifications to the data generating mechanism. For example, the additive causal effect of a dichotomized exposure compares an intervention by which the exposure is truncated below the dichotomization threshold with an intervention by which the exposure is truncated above it [9]. In addition, for most of the variables we discuss below, such interventions are not practical, so the resulting estimates too far removed from practicality.

In this paper we present variable importance measures that can be used to rank a list of both continuous and binary variables in terms of their importance on an outcome. The approach we present differs from other approaches in the literature [1] in that we also address estimation of VIM for continuous variables, as opposed to only binary ones. We use state-of-the-art machine learning coupled with causal inference methods to address VIM estimation and illustrate the use of our methods for analyzing the determinants of death in severe trauma patients.

The paper is organized as follows. We start by describing the problem that motivates the development of the tools presented in this paper, and then move to an introduction of the causal inference tools used to define the variable importance measures. We then present various estimators for the variable importance parameters previously defined and discuss their asymptotic properties; we also briefly describe the super learner [10], an ensemble learner whose asymptotic performance is optimal for prediction. We finish by presenting the details of the data analysis and some concluding remarks.

## Background

Trauma is the leading cause of death between the ages of 1 and 44. The vast majority of these deaths take place quickly and much of the initial resuscitative and decision-making action takes place in the first minutes to hours after injury [11,12]. In addition, it is clear that as patients progress through their initial resuscitation, the relative attention paid to different physiologic and biologic parameters and indeed the interventions themselves are dynamic. Different variables are important and drive future outcome in the first few minutes after injury than at 24 hours when a patient has survived long enough to receive large volume resuscitation, operative intervention and ICU care. While these dynamics are intuitive, most practitioners do not have the ability to know which variables are important at any given time point. As a result, often the same vital signs and markers are followed throughout the patient’s hospital course independent of whether they are currently relevant. This results in practitioners who are often left making care decisions without knowledge of the current patient physiologic state and which parameters are important at that moment. Left with this uncertainty and awash in constantly evolving multivariate data, practitioners make decisions based on clinical gestalt, a few favorite variables, and rules of thumb developed from clinical experience. To aid in prediction, the medical literature is filled with scoring systems and published associations between these variables (physiology, biomarker, demographic, etc.) and outcomes of interest [13–17]. While numerous, these published statistical associations, given the reported methodology, often report misspecified and overfit models. In addition most of these statistical predictive models do not account for the rapidly changing dynamics of a severely injured patient, and fail to take into account the statistical issues discussed in the previous paragraphs. An ideal system would mimic the clinical decision making of an experienced practitioner by providing dynamic prediction (changing prediction at iterative time points) while evaluating the dynamic importance of each variable over time [18]. This then would mimic the implicit understanding a clinician brings to a patient where it is clear that the necessary focus of care must change over time.

### Trauma Data

The data that motivated the methods developed in this paper were collected as part of the Activation of Coagulation and Inflammation in Trauma (ACIT, see e.g., [19–21]) study, which is a prospective cohort study of severe trauma patients admitted to a single level 1 trauma center. Several physiological and clinical measurements were recorded at several time points for each patient after arrival to the emergency room. These variables include demographic variables (e.g., age, gender, etc.), baseline risk factors (e.g., asthma, chronic lung disease, Glasgow coma scale, diabetes, injury mechanism, injury severity score, etc.), longitudinally measured variables that account for the patient’s treatment and health status history (e.g., respiratory and heart rate, platelets, coagulation measures like prothrombin time and INR, activated protein C, etc.), and an indicator of the occurrence of death at each time interval. Because these data are often collected in a high-stress environment, it is common that some variables are missing for some patients at a given time point. The list of variables we analyzed is presented in S1 Table Table of the Supporting Information.

### Classical Regression Approaches

In order to estimate the effect of a variable *A* on an outcome *Y* controlling for a set of variables *W*, it is common practice among data analysts to estimate the parameter *β* in a parametric regression model *E*(*Y*|*A*,*W*) = *m*(*A*,*W*|*β*) for a known function *m*, for example,

$$\begin{array}{c} E\left(Y|A,W\right)=\beta_{0}+\beta_{1}A+\beta_{2}W. \end{array}$$ (1)

Under model (1), the estimate of *β*_1_ is interpreted as the expected change in *Y* given a change of one unit in *A*:

$$\begin{array}{c} \beta_{1}=E\left\{E\left(Y|A+1,W\right)-E\left(Y|A,W\right)\right\}. \end{array}$$ (2)

Small violations to the assumptions of model (1) (e.g., an interaction term) would yield an estimate of *β*_1_ that cannot be interpreted as in (2). Therefore, in this paper we define parameters in terms of characteristics of the probability distribution of the data under a non-parametric model, as in Equation (2). This practice allows the definition of the parameter of interest independently of (possibly) misspecified parametric models, and avoids dealing with different interpretations of regression parameters under incorrect model specifications.

The causal interpretation of statistical parameters (e.g., Equation (2)) requires additional untestable assumptions about the distribution of counterfactual outcomes under hypothetical interventions. Such counterfactual outcomes may be defined using the potential outcomes framework of a structural equation model (NPSEM [22]). In the remaining of the section we describe the observed data, and define the variable importance measures using an NPSEM. If the assumptions encoded in the NPSEM do not hold, the estimates do not have a causal interpretation and must not be used to make treatment decisions. In that case, there are two main uses of these estimates. First, they can be used as tools for determining the best set of predictors variables by ruling out those whose with a zero non-significant variable importance. Second, they may be used as a tool for formulating causal hypothesis that may be tested in a subsequent randomized study or in an observational study in which the necessary causal assumptions are met.

## Methods

### Notation

Assume that observations on each patient are recorded at times *t*_0_,*t*_1_,…,*t*_*J*_, where *t*_0_ = 0, and let *T* denote the time of death of a patient. The observed data for each patient is given by the random variable

$$\begin{array}{c} O=(L_{0},C_{1},L_{1},Y_{1},\ldots ,C_{J},L_{J},Y_{J}), \end{array}$$

where *L*_0_ denotes a set of baseline variables recorded at admission to the hospital, *L*_*j*_ = (*L*_*j*1_,…,*L*_*jK*_) denotes a set of variables measured at time *t*_*j*_, *C*_*j*_ = (*C*_*j*1_,…,*C*_*jK*_) where *C*_*jk*_ denotes an indicator of missingness of *L*_*jk*_, and *Y*_*j*_ = *I*(*t*_*j*_ < *T* ≤ *t*_*j*+1_) denotes an indicator of death occurring in the interval (*t*_*j*_,*t*_*j*+1_], for *j* = 0,…,*J*−1. Once death occurs the random variables in the remaining time points of the vector *O* become degenerate so that this structure is well defined.

For the analysis of the ACIT data we have classified the variables *L*_*jk*_ in two non-mutually exclusive categories: baseline and treatment variables. Baseline variables (*L*_0_) are causally related to the outcome but can seldom be manipulated by the physician and are rarely of interest as possible care targets. Although baseline variables are not of interest in themselves, controlling for them is crucial when estimating the effect of treatment variables, which are often longitudinal variables that represent possible targets for clinical care. The label of each variable according to this classification is shown in S1 Table Table of the Supporting Information.

We define VIM measures in terms of the effect of *L*_*jk*_ on *Y*_*j*^′^_, for all *j* ^′^ ≥ *j* and for all *k*. That is, we are interested in importance of a variable recorded at time point *t*_*j*_ on the hazard of death in each of the subsequent time intervals (*t*_*j*_,*t*_*j*+1_],…,(*t*_*J*−1_,*t*_*J*_]. This approach has the advantage that VIM can be seen as a dynamic process in which the factors that are decisive for developing/predicting a clinical outcome change as a function of time.

### Causal model

We encode the assumptions necessary to make causal claims in terms of the non-parametric structural equation model [22]:

$$\begin{array}{ccc} L_{0} & = & f_{L_{0}}\left(U_{L_{0}}\right)\;\\ C_{jk} & = & f_{C_{jk}}\left(C_{j-1},L_{j-1},L_{0},U_{C_{j}}\right)\;j=1,\ldots ,J;\;k=1,\ldots ,K\\ L_{jk} & = & C_{jk}f_{L_{jk}}\left(C_{j-1},L_{j-1},L_{0},U_{L_{j}}\right)\;j=1,\ldots ,J;\;k=1,\ldots ,K\\ Y_{j} & = & f_{Y_{j}}\left({\bar{C}}_{j},{\bar{L}}_{j},L_{0},U_{Y_{j}}\right)\;j=1,\ldots ,J, \end{array}$$ (3)

where, for a random variable *X*, *f*_*X*_ denotes an unknown but fixed function, *U*_*X*_ denotes all the unmeasured factors that are causally related to *X*, and ${\bar{X}}_{j}=(X_{1},\ldots ,X_{j})$ denotes the history of *X* up until time *t*_*j*_. As pointed out by [22], this model assumes that the data *O* for each patient are generated by the mechanistic process implied by the functions *f*_*X*_*j*__ with a temporal order dictated by the ordering of the time points *t*_*j*_. In addition, this NPSEM encodes two important conditional independence assumptions:

$$\begin{array}{ccc} L_{jk} & ⊥\;\;\;\;\;⊥ & L_{jk^{*}}|\left(L_{0},L_{j-1}\right)\;\forall j,\;k^{*}\neq k, \end{array}$$ (4)

$$\begin{array}{ccc} L_{jk} & ⊥\;\;\;\;\;⊥ & {\bar{L}}_{j-2}|\left(L_{0},L_{j-1}\right)\;\forall j,\;k. \end{array}$$ (5)

Assumption (4) means that the variables *L*_*jk*_ at time *t*_*j*_ are drawn simultaneously as a function of the past only, and that, given the past, contemporary variables do not interact with each other. This is an assumption that must be taken with caution since the causal structure of the relation between contemporary measurements of physiological characteristics of trauma patients is not well understood yet. Assumption (5) is a standard Markov independence assumption stating that *L*_*jk*_ is independent on covariate past conditional on the most recent measurements *L*_*j*−1_ and baseline *L*_0_.

**Fig 1:**
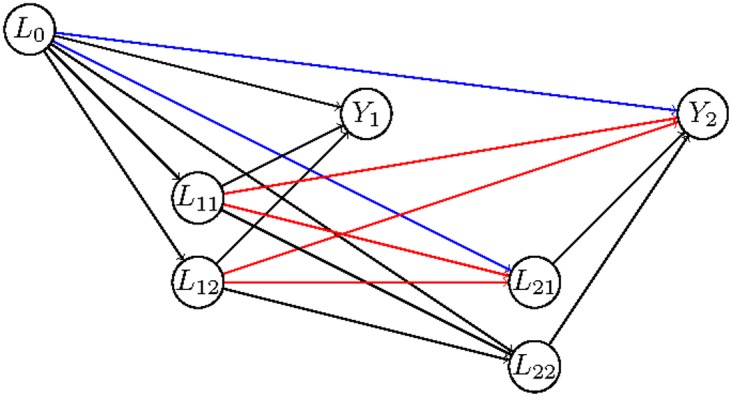
Directed acyclic graph, the arrows in blue and red denote the relations that confound the causal effect of *L*_21_ on *Y*_2_.

As a consequence of these assumptions, the problem of estimating the causal effect of each *L*_*jk*_ on each *Y*_*j*^′^_ for *j* ^′^ ≥ *j* can be seen as a series of cross-sectional problems as follows. Note that *L*_jk*_ for k* ≠ *k* are not confounders of the causal relation between *L*_*jk*_ and *Y*_*j*^′^_. To illustrate this, consider the NPSEM encoded in the directed acyclic graph of Fig. 1, in which for simplicity we assume that all covariates are observed (i.e., *C* variables are not present) and that *J* = *K* = 2. It stems from the graph that the variable *L*_22_ plays no role as a confounder of the causal effect of *L*_21_ on *Y*_2_. The problem of variable importance for these data can thus be transformed into a series of cross-sectional problems as follows. For each patient still at risk at *t*_*j*^′^_, denote

$$\begin{array}{ccc} A & \equiv & L_{jk}\\ C & \equiv & C_{jk}\\ W & \equiv & \left(L_{0},C_{j-1},L_{j-1}\right)\\ Y & \equiv & Y_{j^{'}}, \end{array}$$ (6)

and

$$\begin{array}{c} \begin{array}{c} \bar{Q}\left(A,C,W\right)\equiv E(Y|A,C,W),\;g(A|C,W)\equiv P(A|C,W) \end{array}\\ \begin{array}{c} \phi (C|W)\equiv P(C|W),\;Q_{W}\left(W\right)\equiv P\left(W\right). \end{array} \end{array}$$

Without loss of generality we assume that the variable *A* is either binary or continuous in the interval (0,1). For fixed *j*, *j* ^′^ ≥ *j*, and *k*, and for each patient still at risk at *t*_*j*^′^_, using the notation introduced in (6), it suffices to consider the following simplified NPSEM:

$$\begin{array}{c} W=f_{W}\left(U_{W}\right),\;C=f_{C}\left(W,U_{C}\right),\;A=Cf_{A}\left(W,U_{A}\right),\;Y=f_{Y}\left(A,C,W,U_{Y}\right), \end{array}$$ (7)

where the *U* variables denote all the exogenous, unobserved factors associated to each of the observed variables, and the functions *f* are deterministic but unknown and completely unspecified. Some additional consequences of NPSEM (7) are:

1. The missingness indicator *C* is allowed to depend on the covariates measured in the previous time point. In this way we take into account that a variable can be missing as a result of the previous health status of the patient, and also that it can be correlated with previous missingness indicators. Other approaches to handle missing data such as multiple imputation may be used here, each requiring their own assumptions. However, we favor this approach because i) it is embedded within the same causal inference framework in which we define the VIM parameters, and ii) the validity of the results does not depend on the correct specification of a multiple imputation model.
2. Missingness is informative. A patient’s missingness indicator *C* is allowed to affect the way *Y* is generated, therefore acknowledging that missingness can contain information about the health outcome (e.g., sicker patients who die earlier might be more likely to have missing values because information stops being recorded during life-threatening situations).

We now define the variable importance for continuous and binary outcomes separately.

#### Continuous Variables

Consider an intervened system in which the variables are generated by the following system of equations

$$\begin{array}{ccc} W & = & f_{W}\left(U_{W}\right)\\ C^{I} & = & 1\\ A^{I} & = & f_{A}\left(W,U_{A}\right)+\delta \\ Y^{I} & = & f_{Y}\left(A^{I},C^{I},W,U_{Y}\right), \end{array}$$ (8)

which, for a small positive *δ*, can be interpreted as a model in which there is no missingness, and the distribution of the exposure variable *A* ^*I*^ is shifted to the right by *δ* units. This type of intervention has been previously discussed in the literature [23], and belongs to a wider class of interventions known as stochastic interventions [24–26]. The parameter *E*(*Y* ^*I*^)−*E*(*Y*) can be causally interpreted as the expected reduction in mortality rate gained by an increase of *δ* units in the variable *A* for each patient. Since the counterfactual data *O* ^*I*^ = (*W*,*C* ^*I*^,*A* ^*I*^,*Y* ^*I*^) are not observed, *E*(*Y* ^*I*^) is not estimable without further untestable assumptions. Under the randomization assumption [22, 27]

$$\begin{array}{c} \left(C,A\right)⊥Y^{I}|W, \end{array}$$ (9)

and the positivity assumption

$$\begin{array}{c} g_{0}\left(A|W\right)>0,\;\;\text{and}\;\;\phi_{0}\left(1|W\right)>0\;\;\text{for}\;\text{all}\;A\;\text{and}\;W, \end{array}$$ (10)

the expectation *E*(*Y* ^*I*^) is identified as $E(Y^{I})=E_{W}E\{\bar{Q}(A+\delta ,C,W)|C=1,W\}$, and the parameter of interest is defined as

$$\begin{array}{c} \Psi_{c}\left(\bar{Q},Q_{W},g\right)\equiv E_{W}E_{g}\left\{\bar{Q}\left(A+\delta ,1,W\right)|C=1,W\right\}-E\left(Y\right). \end{array}$$ (11)

A proof of this result under the randomization assumption is presented in [23]. That proof follows the arguments for identification of general causal parameters given in [22], who provides a unified framework for identification of counterfactual parameters as function of the observed data generating mechanism.

#### Binary Variables

For binary variables, following the structural causal model described in (7), the VIM parameter is defined according to the following intervened system:

$$\begin{array}{ccc} W & = & f_{W}\left(U_{W}\right)\\ C^{I} & = & 1\\ A^{I} & = & \left\{\begin{array}{cc} 1 & \text{with}\;\text{probability}\;g(1|1,W)+\delta \\ 0 & \text{with}\;\text{probability}\;g(0|1,W)-\delta \end{array}\right.\\ Y^{I} & = & f_{Y}\left(A^{I},C^{I},W,U_{Y}\right), \end{array}$$

where 0 < *δ* < sup_*w*_ *g*(0|1,*w*) is a user-given value. Under randomization assumption (9), and the positivity assumption

$$\begin{array}{c} 0<g_{0}\left(1|W\right)<1,\;\;\text{and}\;\;\phi_{0}\left(1|W\right)>0\;\;\text{for}\;\text{all}\;W, \end{array}$$ (12)

the expectation of *Y* ^*I*^ is identified as a function of the observed data generating mechanism as $E(Y^{I})=E_{W}E\{\bar{Q}(A,C,W)|C=1,W\}+\delta \{E[\bar{Q}(1,1,W)-\bar{Q}(1,0,W)]\}$, and the parameter of interest is defined as

$$\begin{array}{c} \Psi_{b}\left(\bar{Q},Q_{W},g\right)\equiv E_{W}E_{g}\left\{\bar{Q}\left(A,C,W\right)|C=1,W\right\}+\delta \left\{E\left[\bar{Q}\left(1,1,W\right)-\bar{Q}\left(1,0,W\right)\right]\right\}-E\left(Y\right), \end{array}$$ (13)

The true values of Ψ_*c*_ and Ψ_*b*_ will be denoted *ψ*_*c*,0_ and *ψ*_*b*,0_, respectively.

#### Comparability

Since the previous variable importance will be used to provide a ranking of the variables mixing continuous and binary ones, we provide a heuristic argument that they are comparable up to first order. First of all, note that, under the appropriate differentiability assumptions, for continuous *A* we have

$$\begin{array}{c} \Psi_{c}\left(\bar{Q},Q_{W},g\right)\approx E_{W}\left\{\bar{Q}\left(A,1,W\right)|C=1,W\right\}+\delta \frac{d}{d\delta }E_{W}E\left\{\bar{Q}\left(A+\delta ,1,W\right)|C=1,W\right\}{|}_{\delta =0}. \end{array}$$ (14)

This expression and (13) both have the form *a*+*δ*×*b*, where *b* can be seen as the appropriate slope of $E\{\bar{Q}(A,C,W)\}$ as a function of *A*.

To illustrate this, let us consider the following scenarios. First, consider a continuous variable *A*_1_ distributed as *Beta*(2,2)×0.8+0.1, and a binary variable *A*_2_ distributed as *Ber*(*A*_1_), where *Ber*(*p*) is the Bernoulli distribution with probability *p*. If the outcome is continuous, with no missingness, and $\bar{Q}(A_{1},A_{2})=A_{1}+A_{2}$, it is straightforward to see that *ψ*_*c*,0_ = *ψ*_*b*,0_ = *δ*, for any 0 < *δ* < 0.1. Equality of these parameters is a consequence of the comparability argument above, and the fact that the approximation (14) is exact for linear $\bar{Q}$. Consider now a nonlinear case, with *Y* binary distributed as *Ber*(expit(*A*_1_+*A*_2_)). In this case, Monte Carlo computation of the integrals involved yields *ψ*_*c*,0_ = 0.0019 and *ψ*_*b*,0_ = 0.0020 for *δ* = 0.01, providing an example of the comparability argument described above.

In the following sections we discuss doubly robust estimation methods for these parameters for continuous and binary variables.

### VIM estimation

In order to define semi-parametric VIM estimates that have optimal asymptotic properties we first need to talk about the efficient influence function. The efficient influence function is a known function *D* of the data *O* and *P*_0_, and is a key element in semi-parametric efficient estimation, since it defines the linear approximation of all efficient regular asymptotically linear estimators [28]. This means that the variance of the efficient influence function provides a lower bound for the variance of all regular asymptotically linear estimators, analogously to the Cramer-Rao lower bound in parametric models. The efficient influence functions of parameters Ψ_*c*_ and Ψ_*b*_ are presented in Section S1 TMLE Algorithm of the Supporting Information.

We use targeted minimum loss based estimators (TMLE)[29,30] of the parameters Ψ_*c*_ and Ψ_*b*_. TMLE is a substitution/plug-in estimation method that, given initial estimators $\left({\bar{Q}}_{n},Q_{W,n},g_{n}\right)$ of $\left(\bar{Q},Q_{W},g\right)$, finds updated estimators $\left({\bar{Q}}_{n}^{*},Q_{W,n}^{*},g_{n}^{*}\right)$ to define the estimator of Ψ as

$$\begin{array}{c} \psi_{n}=\Psi \left({\bar{Q}}_{n}^{*},Q_{W,n}^{*},g_{n}^{*}\right). \end{array}$$

TMLE is an estimation method that enjoys the best properties of both G-computation estimators [31] and the estimating equation methodology (see e.g., [32, 33]). On one hand, TMLE is similar to G-computation estimators (e.g., $\Psi ({\bar{Q}}_{n},Q_{W,n},g_{n})$) in that it is a plug-in estimator, and therefore produces estimates that are always within the range of the parameter of interest (e.g., it is always in the interval [0, 1] if the estimand is a probability). On the other hand, under regularity conditions and consistency of $\left({\bar{Q}}_{n},g_{n},\phi_{n}\right)$, it is asymptotically linear with influence function equal to the efficient influence function:

$$\begin{array}{c} \psi_{n}-\psi_{0}=\sum_{i=1}^{n}D\left(P_{0}\right)\left(O_{i}\right)+o_{P}\left(1/\sqrt{n}\right). \end{array}$$

As a consequence, TMLE has the following properties:

- It respects the known bounds of the target parameter.
- It is efficient if ${\bar{Q}}_{n},g_{n}$, and *ϕ*_*n*_ are consistent for ${\bar{Q}}_{0},g_{0}$, and *ϕ*_0_, respectively.
- It is consistent if either ${\bar{Q}}_{n}$ or both *g*_*n*_ and *ϕ*_*n*_ are consistent. This property is referred to as double robustness.
- It is more robust to empirical violations of the positivity assumptions (10) and (12).

In Section S1 TMLE Algorithm of the Supporting Information we describe an iterative procedure that transforms the initial estimates ${\bar{Q}}_{n}$ and *g*_*n*_ into targeted estimates ${\bar{Q}}_{n}^{*}$ and $g_{n}^{*}$ such that $\Psi ({\bar{Q}}_{n}^{*},g_{n}^{*},Q_{W,n}^{*})$ is a TMLE of $\Psi ({\bar{Q}}_{0},g_{0},Q_{W,0})$, and discuss in more detail the properties of the TMLE.

#### Estimating equation (EE), Gcomp/IPMW, and unadjusted estimators

For comparison, we compute three additional estimates of the VIM. The first estimator, based on the estimating equation (EE) methodology, is an estimator that uses the efficient influence function of the parameter in order to define the estimator as the solution of the corresponding estimating equation. Because the EE is also asymptotically linear with influence function equal to the efficient influence function, it is consistent and asymptotically efficient under regularity and consistency conditions on $\left({\bar{Q}}_{n},Q_{W,n},g_{n}\right)$. However, the estimating equation that defines the EE may not have a solution in the parameter space, in which case the EE does not exist. The second estimator, a mixture of the G-computation formula and the inverse probability of missingness weighted estimator IPMW (Gcomp/IPMW) represents a choice that could have been made in common practice in statistics. The Gcomp/IPMW estimator uses initial estimators *ϕ*_*n*_ and ${\bar{Q}}_{n}$ of *ϕ*_0_ and ${\bar{Q}}_{0}$ obtained through step-wise regression, and is defined as

$$\begin{array}{ccc} \psi_{c,n,GI} & = & \frac{1}{n}\sum_{i=1}^{n}\left\{\frac{C_{i}}{\phi_{n}\left(W_{i}\right)}{\bar{Q}}_{n}\left(A_{i}+\delta ,1,W_{i}\right)-Y_{i}\right\}\\ \psi_{b,n,GI} & = & \frac{1}{n}\sum_{i=1}^{n}\left\{\frac{C_{i}}{\phi_{n}\left(W_{i}\right)}{\bar{Q}}_{n}\left(A_{i},1,W_{i}\right)+\delta \left[{\bar{Q}}_{n}\left(1,1,W_{i}\right)-{\bar{Q}}_{n}\left(0,1,W_{i}\right)\right]-Y_{i}\right\}, \end{array}$$

for Ψ_*c*_ and Ψ_*b*_, respectively. This estimator is consistent only if both the model for *ϕ*_0_ and the model for ${\bar{Q}}_{0}$ have been correctly specified. The unadjusted estimator is identical to the Gcomp/IPMW estimator but including only the intercept term in the vector *W*.

Since the consistency of the initial estimators of ${\bar{Q}}_{0}$, *g*_0_ and *ϕ*_0_ is key to attain estimators with optimal statistical properties (i.e., consistency and efficiency), we carefully discuss the construction of such estimators in the next subsection. In particular, the next subsection deals with the construction of an estimator for ${\bar{Q}}_{0}$, the predictor of death in our working example.

### Prediction via SuperLearner

As explained in the previous section, the consistency of the initial estimators ${\bar{Q}}_{n}$, *g*_*n*_ and *ψ*_*n*_ determine the statistical properties of the estimators of *ψ*_*c*,0_ and *ψ*_*b*,0_. Common practice in statistics involves the estimation of models like

$$\begin{array}{c} \mathrm{logit}\bar{Q}\left(A,W\right)=\beta_{0}+\beta_{1}A+\beta_{2}W+\beta_{3}AW. \end{array}$$ (15)

This approach that has gained popularity among researchers in epidemiology and biostatistics, partly because of the analysis of its statistical properties requires simple mathematical methods, and partly because it is readily available in statistical software. Nevertheless, as it is also well known among their users, parametric models of the type described by (15) are rarely correct, and their choice is often based on their continence and other subjective criteria. This practice leads to estimator whose usefulness is highly questionable given that the assumptions it entails (linearity, normality, link function, etc.) will rarely be theoretically supported.

In this paper we use the super learner [10] for estimation of ${\bar{Q}}_{0}$, *g*_0_, and *ψ*_0_. Super learner is a methodology that uses cross-validated risks to find an optimal combination of a list of user-supplied estimation algorithms. One of its most important theoretical properties is that its solution converges to the oracle estimator (i.e., the candidate in the library that minimizes the loss function with respect to the true probability distribution), thus providing the closest approximation to the real data generating mechanism. Proofs and simulations regarding these and other asymptotic properties of the super learner can be found in [34] and [35].

To implement the super learner predictor it is necessary to specify a library of candidate predictors algorithm. In the case of the conditional expectations ${\bar{Q}}_{0}$, *ϕ*_0_, and *g*_0_ for binary *A*, the candidates can be any regression or classification algorithm. Examples include random forests, logistic regression, *k* nearest neighbors, Bayesian models, etc. For estimation of the conditional densities *g*_0_ we also use the super learner, with candidates given by several histogram density estimators, which yields a piece-wise constant estimator of the conditional density. The choice of the number of bins and their location is indexed by two tuning parameters. The implementation of this density estimator is discussed in detail in [36], and is omitted in this paper.

### Details of Data Analysis

The sample size was *n* = 918 patients, and measurements of the variables described in S1 Table Table of the Supporting Information were taken at 6, 12, 24, 48, and 72 hours after admission to the emergency room.

The main objective of the study was the construction of prediction models for the risk of death of a patient in a certain time interval given the variables measured up to the start of the interval, as well as the definition and estimation of VIM measures that provide an account of the longitudinal evolution of the relation between these physiological and clinical measurements and the risk of death at a certain time point.

The data set was partitioned in 6 different data sets according to the time intervals defined by the time points in which measurements were taken, each of these 6 datasets contained only the patients that were at risk of death (alive) at the start of the time interval. Each of the continuous covariates was rescaled by subtracting the minimum and dividing by the range so that all of the covariates range between zero and one. The methods described in the previous sections were applied to each variable in each of these datasets.

The candidate algorithms for prediction of death used in the super learner predictor are:

- Logistic regression with main terms (GLM)
- Stepwise logistic regression (SW)
- Bayesian logistic regression (BLR) [37]
- Generalized additive models (GAM) [38]
- MARS (MARS) [39]
- Sample mean (MEAN).

The first three represent common practice in epidemiology and statistics, and the GAM and MARS algorithms intend to capture nonlinearities in relations between the data. The sample mean is included for contrast.

The super learner is a “black box” prediction algorithm, constructed to minimize the prediction error. As such, the coefficients of each candidate do not have any meaningful interpretation. To see this, consider a hypothetical situation in which two prediction candidates provide highly correlated predictions. In that case, the coefficients of the two candidates in the library will be highly variable, but the prediction error and the super learner will not be affected by such variability.

## Results

Table 1 shows the coefficients of each candidate algorithm in the super learner predictor of $E(Y_{j}|{\bar{L}}_{j},{\bar{C}}_{j},L_{0})$. The variability in these coefficients shows that no single algorithm is optimal for prediction at each time point, and that each algorithm describes certain features of the data generating mechanism that the others are not capable of unveiling, advocating for the need of an automated method to choose between them.

**Table 1:** Coefficients in the Super Learner.

	0–6hr	6–12hr	12–24hr	24–48hr	48–72hr	72+hr
GLM	0.0000	0.0000	0.0000	0.0318	0.0259	0.0000
SW	0.0000	0.1889	0.0000	0.0000	0.2073	0.1787
BGLM	0.3318	0.0586	0.1049	0.1329	0.0313	0.2750
GAM	0.5118	0.7525	0.8951	0.8353	0.7201	0.2487
MARS	0.1563	0.0000	0.0000	0.0000	0.0154	0.1298
MEAN	0.0000	0.0000	0.0000	0.0000	0.0000	0.1678

**Fig 2:**
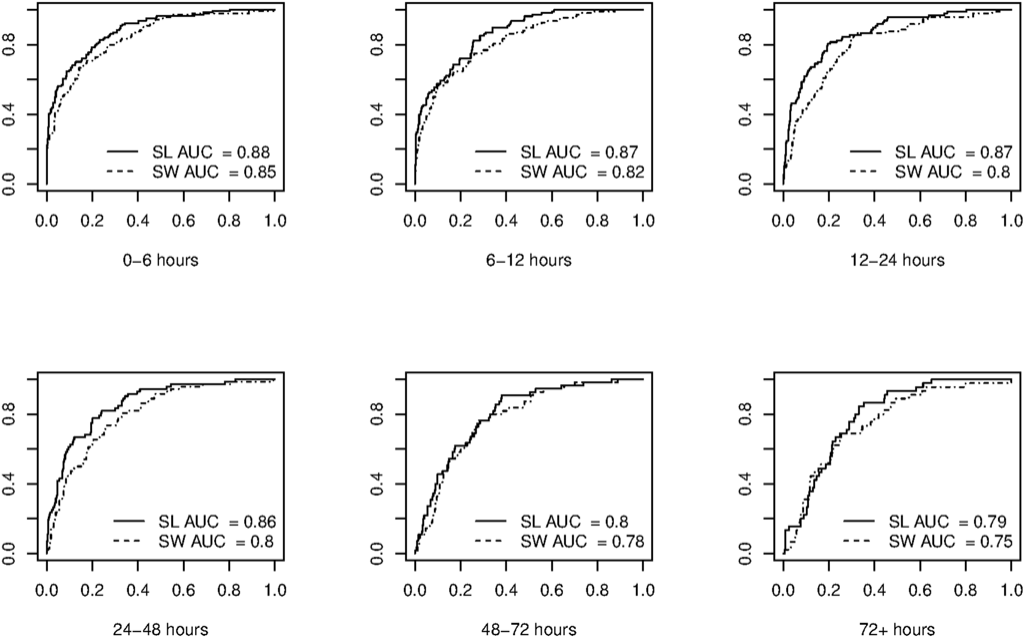
ROC curves of cross-validated prediction for the super learner (SL) and the logistic step-wise regression (SW), for different time intervals.

Fig. 2 presents the ROC curves for the cross-validated super learning predictions of death, as well as the cross-validated predictions based on a logistic model with AIC-based stepwise selection of variables, for comparison with common practice. The super learner prediction method outperforms the stepwise prediction in all cases, with AUC ROC (area under the ROC curve) differences ranging from 0.02 to 0.07. Though this differences might be small, an interpretation of their meaning reveals the clinical relevance of a slight improvement in prediction. The AUC ROC can be interpreted as the proportion of times that a patient who dies obtains a higher prediction score than a patient who survives. In practice, an AUC ROC difference of 0.02 means that in 100 pairs of patients (pairs formed by one patient who dies and one who does not) the super learner, on average, classifier correctly classifies two pairs more than the step-wise classifier, which could potentially lead to live-saving treatments for these two patients.

The VIM-TMLE measures that were significant at 0.05 were ranked according to their magnitude. Table 2 presents the first five (whenever five or more were significant) most important variables for prediction of death at each time interval, according to the TML estimator previously introduced. Recall that all the continuous variables were re-scaled between zero and one; the value *δ* = 0.01 was used for all the estimates. The interpretation of the values in the first row of Table 2, for example, is that if APC were to increase by 1% for every patient, the mortality rate in the first time interval would, on average, increase by 2%. The TMLE and the EE produced generally similar results, whereas the Gcomp/IPMW estimator produced results that are somewhat different and with greater estimated variability. Note that several of the Gcomp/IPMW point estimates coincide with the TMLE and EE, but their p-values are generally larger. This could be due to the fact that the TMLE and EE are locally efficient estimators, and therefore provide more powerful hypothesis tests. In light of the superior theoretical properties of the TMLE and EE, we prefer to rely on estimates obtained through these methods.

**Table 2:** VIM estimates for the most important variables for prediction of death at each time interval according to TML estimate (p-values in parentheses and truncated at 0.001).

Time of death	Var. Name	Var. Time	TMLE	EE	G-comp/IPMW	Unadjusted
0–6 hrs.	APC	00	0.0205(0.023)	0.0183(0.043)	0.0235(0.386)	0.1063(< 0.001)
	INR	00	0.0216(< 0.001)	0.0193(0.002)	−0.0011(0.722)	0.0345(< 0.001)
	PT	00	0.0248(< 0.001)	0.0248(< 0.001)	−0.0011(0.698)	0.0400(< 0.001)
	ISS	00	−0.0314(< 0.001)	−0.0319(< 0.001)	−0.0242(< 0.001)	−0.0244(0.002)
6–12 hrs.	SBP	00	0.0041(0.010)	0.0041(0.011)	0.0020(0.108)	0.0340(< 0.001)
	PT	00	0.0076(0.001)	0.0074(0.001)	0.0019(0.426)	0.0376(< 0.001)
	BDE	00	0.0098(0.028)	0.0114(0.011)	0.0120(0.004)	0.0796(< 0.001)
	FV	06	−0.0139(0.047)	−0.0394(0.018)	−0.0394(0.141)	0.0199(0.349)
	ATIII	06	−0.0160(0.026)	−0.0323(0.009)	−0.0434(0.072)	0.0199(0.340)
12–24 hrs.	SBP	00	0.0031(0.034)	0.0030(0.037)	0.0018(0.220)	0.0317(< 0.001)
	PT	00	0.0051(0.025)	0.0048(0.031)	0.0019(0.377)	0.0357(< 0.001)
	FV	06	−0.0134(0.050)	−0.0246(0.008)	−0.0327(0.562)	0.0276(0.140)
	DDIM	00	0.0140(0.042)	0.0134(0.054)	0.0142(0.331)	0.0955(< 0.001)
	PC	06	−0.0224(0.001)	−0.0394(0.008)	−0.0286(0.349)	0.0262(0.221)
24–48 hrs.	PT	00	0.0080(< 0.001)	0.0080(< 0.001)	0.0026(0.124)	0.0313(< 0.001)
	DDIM	00	0.0134(0.026)	0.0133(0.028)	0.0144(0.504)	0.0770(< 0.001)
	ISS	00	−0.0229(< 0.001)	−0.0232(< 0.001)	−0.0211(< 0.001)	−0.0155(0.020)
	HR	12	0.0346(< 0.001)	0.0136(0.118)	−0.0030(0.922)	0.0570(< 0.001)
	APC	00	0.0429(< 0.001)	0.0432(< 0.001)	0.0226(0.310)	0.0761(< 0.001)
48–72 hrs.	CREA	00	0.0028(0.030)	0.0028(0.030)	0.0007(0.301)	0.0204(< 0.001)
	PT	00	0.0089(< 0.001)	0.0089(< 0.001)	0.0017(0.210)	0.0241(< 0.001)
	DDIM	12	0.0140(0.049)	0.0122(0.095)	0.0481(0.885)	0.0605(< 0.001)
	PC	06	−0.0164(0.012)	−0.0190(0.010)	−0.0252(0.075)	0.0381(0.035)
	RR	24	0.0187(0.002)	0.0148(0.012)	0.0057(0.749)	0.0644(< 0.001)
72+ hrs.	CREA	00	0.0027(0.002)	0.0027(0.002)	0.0007(0.291)	0.0168(< 0.001)
	ISS	00	−0.0142(0.005)	−0.0149(0.003)	−0.0145(0.008)	−0.0085(0.124)
	PTT	00	0.0220(< 0.001)	0.0219(< 0.001)	0.0017(0.012)	0.0220(< 0.001)

## Discussion

In this paper we address the problem of estimating variable importance parameters for longitudinal data that are subject to missingness. We present variable importance parameters that have a clear interpretation either as purely statistical parameters or as causal effects, depending on the assumptions about the data generating mechanism that the researcher is willing to make. These are important characteristics that advance the field in various fronts. First, unlike VIMs derived from machine learning and data-adaptive predictors (e.g., random forests), the VIMs defined in this paper have a concise definition as statistical parameters, which allowed the study of its asymptotic statistical properties and ultimately led to the construction of estimators with desirable statistical properties like consistency, efficiency, and asymptotic normality. Second, the assumptions required to give a causal interpretation to statistical parameters are often concealed, and the language used attempts to imply causal relations without clearly stating the necessary assumptions. The framework we present endows the user with the necessary tools to decide whether it is correct or not to interpret the estimates in terms of causal relations. Additionally, the parameters that we present have a purely statistical interpretation as a measure of conditional dependence, interpretation that must be used when there is not enough knowledge about the causal structure. We provide a methodology that can be used to compare continuous and binary variables in terms of their effect on an outcome, guaranteeing that the results are mathematically comparable.

We illustrate the use of the methods through the analysis of the drivers of recovery after sever trauma. These analyses provide a significant contribution to the field of trauma injury, by bringing state-of-the-art statistical methods to a field in which the large dimensionality of the problem constitutes a limiting factor for understanding the intricate relations between the variables involved. We propose a “black-box” prognosis algorithm (super learner) that can take into account the complexity of the problem, and represents an alternative to the scoring methods based on rules of thumb that are currently used in this setting. The results of the variable importance analysis corroborate the hypothesis that recovery after severe trauma is a dynamic process in which the decisive factors change over time, and provides provisional answers to various questions about recovery after severe trauma. Because there is not certainty that the structural causal assumptions required are met, the estimated VIMs can only be used as predictive performance measures and used to postulate hypothesis about causal relations that can be tested in more carefully designed studies. An additional advantage of a more carefully designed study is the possibility of performing a detailed comparison of the trajectories of each variable, using data that is not subject to missingness, or in which the amount of missingness is controlled.

We proposed a TMLE and an estimating equation estimator. Both of these estimators are doubly robust and efficient under certain regularity and consistency conditions of the initial estimators, but the TMLE has the additional advantages of a plug-in estimator. However, we did not observe any relevant difference between them in the illustration example. Various authors [23] have already compared these two estimators through a simulation study under no missingness of the treatment variable, finding no difference between them. We proposed the G-comp/IPMW, an additional estimator that represents an easy alternative to the TMLE or EE. Although we found various differences in the magnitude of the estimates between the TMLE and the G-comp/EE, the main discrepancy was with respect to the standard errors and p-values. We conjecture that these differences are a consequence of the inefficiency of the G-comp/IPMW, which results in hypothesis tests with less power. As discussed in Section S1 TMLE Algorithm of the Supporting Information, the proposed TMLE is defined by an iterative procedure that involves numerical integration of super learning predictions at each step. This represents a drawback of the estimator in terms of scalability when compared to the estimating equation (EE) methodology. Because the estimating equation methodology involves only computation of predicted values from three super learners (outcome, missingness mechanism, and treatment mechanism), and averaging of a function of these predictions, its computational time is expected to be of the order of the computational time of the learners considered in the library.

Finally, given the flexibility of this general approach, and the ability to automate the algorithms, these type of variable importance measures promise great benefit in high dimensional clinical and other longitudinal settings.

## Supporting Information

S1 Table Variables in the ACIT data set. (PDF)

S1 TMLE Algorithm TMLE Algorithm. (PDF)
