# Supplementary material for: Variable Importance and Prediction Methods for Longitudinal Problems with Missing Variables
Source: PLoS One. 2015 Mar 27;10(3):e0120031. doi: 10.1371/journal.pone.0120031 (PMC4376910; doi:10.1371/journal.pone.0120031)
Supplement: S1 Table — (PDF) [file pone.0120031.s001.pdf]

Variable List

The variables analyzed in the ACIT study are presented in Table 1.

Variable	Type	Description
Age	Baseline	Age in years
GCS	Baseline/Treatment	Arrival Glascow Comma Score
ISS	Baseline/Treatment	Injury Severity Score
Asthma	Baseline	Indicator of previous Asthma
COPD	Baseline	Indicator of previous Chronic Obstructive Pulmonary Disease
OCLG	Baseline	Indicator of Other Chronic Lung Disease
CAD	Baseline	Coronary Artery Disease
CHF	Baseline	Congestive Heart Failure
ESRD	Baseline	End Stage Renal Disease
CIRR	Baseline	Cirrhosis
DIAB	Baseline	Diabetes
HPAN	Baseline	Hypoalbuminemia
Gender	Baseline	Gender
MECH	Baseline	Injury mechanism: blunt or penetrating
HR	Treatment	Heart Rate
RR	Treatment	Respiratory Rate
SBP	Treatment	Spontaneous Bacterial Peritonitis
BDE	Treatment	Base Deficit/Excess
BUN	Treatment	Blood Urea Nitrogen
CREA	Treatment	Creatinine
HGB	Treatment	Hemoglobin
HCT	Treatment	Hematocrit
PLTS	Treatment	Platelets
PT	Treatment	Prothrombin Time
PTT	Treatment	Partial Prothrombin Time
INR	Treatment	International Normalized Ratio
FV	Treatment	Factor III
FVIII	Treatment	factor VIII
ATIII	Treatment	Antithrombin III
PC	Treatment	Protein C
DDIM	Treatment	D-Dimer
TPA	Treatment	Tissue Plasminogen Activator
PAI	Treatment	Plasminogen Activator Inhibitor
SEPCR	Treatment	Soluble Endothelial Protein C Receptor
STM	Treatment	Soluble Thrombomodulin
APC	Treatment	Activated Protein C

Table 1. Variables in the ACIT data set
