# Supplementary material for: Variable Importance and Prediction Methods for Longitudinal Problems with Missing Variables
Source: PLoS One. 2015 Mar 27;10(3):e0120031. doi: 10.1371/journal.pone.0120031 (PMC4376910; doi:10.1371/journal.pone.0120031)
Supplement: S1 TMLE Algorithm — (PDF) [file pone.0120031.s002.pdf]

TMLE Algorithm

The efficient influence function of parameters Ψ_c and Ψ_b are given by

$$D_c(\bar{Q}, Q_W, g, \phi)(O) = D_{c1}(\bar{Q}, g, \phi)(O) + D_{c2}(\bar{Q}, g, \phi)(O) + D_{c3}(\bar{Q}, Q_W, g)(O) \quad (1)$$
$$D_b(\bar{Q}, Q_W, g, \phi)(O) = D_{b1}(\bar{Q}, g, \phi)(O) + D_{b2}(\bar{Q}, g, \phi)(O) + D_{b3}(\bar{Q}, Q_W, g)(O), \quad (2)$$

respectively, where

$$\begin{aligned} D_{c1}(\bar{Q}, g, \phi)(O) &= \frac{C}{\phi(1|W)} \frac{g(A-\delta|1, W)}{g(A|1, W)} \{Y - \bar{Q}(A, 1, W)\} \\ D_{c2}(\bar{Q}, g, \phi)(O) &= \frac{C}{\phi(1|W)} \left[\bar{Q}(1, A+\delta, W) - E_g\{\bar{Q}(1, A+\delta, W)|C=1, W\}\right] \quad (3) \\ D_{c3}(\bar{Q}, Q_W, g)(O) &= E_g\{\bar{Q}(1, A+\delta, W)|C=1, W\} - Y - \Psi_c(\bar{Q}, Q_W, g), \end{aligned}$$

and

$$\begin{aligned} D_{b1}(\bar{Q}, g, \phi)(O) &= \frac{C}{\phi(1|W)} \left(\delta \frac{2A-1}{g(A|1, W)} + 1\right) \{Y - \bar{Q}(A, 1, W)\} \\ D_{b2}(\bar{Q}, g, \phi)(O) &= \frac{C}{\phi(1|W)} [\bar{Q}(A, 1, W) - E_g\{\bar{Q}(A, 1, W)|C=1, W\}] \quad (4) \\ D_{b3}(\bar{Q}, Q_W, g)(O) &= \delta\{\bar{Q}(1, 1, W) - \bar{Q}(0, 1, W)\} + E_g\{\bar{Q}(A, 1, W)|C=1, W\} - Y - \Psi_b(\bar{Q}, Q_W, g). \end{aligned}$$

Result **??** provides the conditions under which these estimating equations have expectation zero, therefore leading to consistent, triply robust estimators.

Result 1 *Let D be either D_c or D_b presented in equations (**??**) and (**??**). We have that*

$$E_{P_0}\{D(O|\phi, g, \bar{Q}, \psi_0)\} = 0$$

if either ($\bar{Q} = \bar{Q}_0$ and $\phi = \phi_0$) or ($\bar{Q} = \bar{Q}_0$ and $g = g_0$) or ($g = g_0$ and $\phi = \phi_0$).

Recall that an estimator that solves an estimating equation is consistent if the expectation of the estimating equation equals zero. As a consequence of this result, and under the conditions on $\bar{Q}$, g and ϕ stated in Theorem 5.11 and 6.18 of [**?**], an estimator that solves the efficient influence function D is consistent if either two of the three initial estimators are consistent, and it is efficient if all of them are consistently estimated. Mathematical proofs of the efficiency of these estimators are out of the scope of this paper, but the general theory underlying their asymptotic properties can be found in [**?**], among others.

In order to define a targeted maximum likelihood estimator for ψ_0, we need to define three elements: (1) A loss function $L(Q)$ for the relevant part of the likelihood required to evaluate $\Psi(P)$, which in this case is $Q = (\bar{Q}, g, Q_W)$. This function must satisfy $Q_0 = \arg\min_Q E_{P_0} L(Q)(O)$, where Q_0 denotes the true value of Q; (2) An initial estimator Q_n^0 of Q_0; (3) A parametric fluctuation $Q(\epsilon)$ through Q_n^0 such that the linear span of $\frac{d}{d\epsilon} L\{Q(\epsilon)\}|_{\epsilon=0}$ contains the efficient influence curve $D(P)$ defined by either (**??**) or (**??**), depending on whether A is continuous or binary. These elements are defined below:

Loss Function

As loss function for Q, we consider $L(Q) = L_Y(\bar{Q}) + L_A(g) + L_W(Q_W)$, where $L_Y(\bar{Q}) = Y \log\{\bar{Q}(A, W)\} + (1-Y)\log\{1-\bar{Q}(A, W)\}$, $L_A(g) = -\log g(A|W)$, and $L_W(Q_W) = -\log Q_W(W)$. It can be easily verified that this function satisfies $Q_0 = \arg\min_Q E_{P_0} L(Q)(O)$.

Parametric Fluctuation

Given an initial estimator Q_n^k of Q_0, with components $(\bar{Q}_n^k, g_n^k, Q_{W,n}^k)$, we define the $(k+1)$th fluctuation of Q_n^k as follows:

$$\operatorname{logit} \bar{Q}_n^{k+1}(\epsilon_1)(A,W) = \operatorname{logit} \bar{Q}_n^k(A,W) + \epsilon_1 H_1^k(C,A,W)$$
$$g_n^{k+1}(\epsilon_1)(A|W) \propto \exp\{\epsilon_1 H_2^k(A,W)\} g_n^k(A|W)$$
$$Q_{W,n}^{k+1}(\epsilon_2)(W) \propto \exp\{\epsilon_2 H_3^k(W)\} Q_{W,n}^k(W),$$

where the proportionality constants are so that the left hand side terms integrate to one, for continuous A

$$H_1^k(A,C,W) = \frac{C}{\phi_n(1|W)} \frac{g_n^k(A-\delta|,1W)}{g_n^k(A|,1,W)},$$

for binary A

$$H_1^k(A,C,W) = \frac{C}{\phi_n(1|W)} \left(\delta \frac{2A-1}{g_n^k(A|1,W)} + 1\right),$$

$H_2^k(A,W) = D_2(P^k)(O)$, and $H_3(W) = D_3(P^k)(O)$, with D_2 and D_3 defined as in (**??**) and (**??**). We define these fluctuations using a two-dimensional ϵ with two different parameters ϵ_1 and ϵ_2, though it is theoretically correct to define these fluctuations using any dimension for ϵ, as far as the condition $D(P) \in < \frac{d}{d\epsilon} L\{Q(\epsilon)\}|_{\epsilon=0} >$ is satisfied, where $< \cdot >$ denotes linear span. The convenience of the particular choice made here is clear once the targeted maximum likelihood estimator (TMLE) is defined.

Targeted Maximum Likelihood Estimator

The TMLE is defined by the following iterative process:

1. Initialize $k = 0$.
2. Estimate ϵ as $\epsilon_n^k = \arg\min_\epsilon P_n L\{Q_n^k(\epsilon)\}$.
3. Compute $Q_n^{k+1} = Q_n^k(\epsilon_n^k)$.
4. Update $k = k+1$ and iterate steps 2 through 4 until convergence (i.e., until $\epsilon_n^k = 0$)

First of all, note that the value of ϵ_2 that minimizes the part of the loss function corresponding to the marginal distribution of W in the first step (i.e., $-P_n \log Q_{W,n}^1(\epsilon_2)$) is $\epsilon_2^1 = 0$. Therefore, the iterative estimation of ϵ only involves the estimation of ϵ_1. The kth step estimation of ϵ_1 is obtained by minimizing $P_n(L_Y(\bar{Q}_n^k(\epsilon_1)) + L_A(g_n^k(\epsilon_1)))$, which implies solving the estimating equation

$$S^k(\epsilon_1) = \sum_{i=1}^n \Bigg\{ \left[Y_i - \operatorname{expit}\{\operatorname{logit} \bar{Q}_n^k(A_i,W_i) + \epsilon_1 H_1^k(O_i)\}\right] H_1^k(O_i) + D_2(P_n^k)(O_i) - \frac{\int_{\mathcal{A}} D_2(P_n^k)(Y_i,a,W_i)\ \exp\{\epsilon_1 D_2(P_n^k)(Y_i,a,C_i,W_i)\}\ g_n^k(a|1,W_i)\, d\mu(a)}{\int_{\mathcal{A}} \exp\{\epsilon_1 D_2(P_n^k)(Y_i,a,C_i,W_i)\}\ g_n^k(a|1,W_i)\, d\mu(a)} \Bigg\} \quad (5)$$

where

$$D_2(P_n^k)(O) = \bar{Q}_n^k(A+\delta,1,W) - \int_{\mathcal{A}} \bar{Q}_n^k(a+\delta,1,W) g_n^k(a|1,W)\, d\mu(a).$$

The TMLE of ψ_0 is defined as $\psi_n \equiv \lim_{k\to\infty} \Psi(P_n^k)$, assuming this limit exists. In practice, the iteration process is carried out until convergence in the values of ϵ_k is achieved, and an estimator Q_n^* is obtained. Under the conditions of Theorem 2.3 of [**?**], a conservative estimator of the variance of ψ_n is given by

$$\frac{1}{n}\sum_{i=1}^n D^2(\bar{Q}_n^*, Q_{W,n}, g_n^*, \phi_n)(O_i).$$
